# The Retraction Penalty: Evidence from the Web of Science

**DOI:** 10.1038/srep03146

**Published:** 2013-11-06

**Authors:** Susan Feng Lu, Ginger Zhe Jin, Brian Uzzi, Benjamin Jones

**Affiliations:** 1 Simon School of Business, University of Rochester; 2 University of Maryland & NBER; 3 Kellogg School of Management, Northwestern University & NICO; 4 Kellogg School of Management, Northwestern University & NBER

## Abstract

Scientific articles are retracted at increasing rates, with the highest rates among top journals. Here we show that a single retraction triggers citation losses through an author's prior body of work. Compared to closely-matched control papers, citations fall by an average of 6.9% per year for each prior publication. These chain reactions are sustained on authors' papers (a) published up to a decade earlier and (b) connected within the authors' own citation network by up to 4 degrees of separation from the retracted publication. Importantly, however, citation losses among prior work disappear when authors self-report the error. Our analyses and results span the range of scientific disciplines.

The science community regularly experiences instances of major scientific mistakes or misconduct. Prominent examples include retracted claims about cloning human embryos and harvesting their stem cells, a claimed link between the MMR vaccine and autism, and claims about super-conducting plastics that misled scientists for years across many top physics laboratories^1^,^2^. In recent years, a Nobel Prize winner has retracted 3 influential papers on the olfactory system, a Harvard evolutionary biologist resigned over scientific misconduct, and a prominent psychologist at Tilburg University admitted to pervasive falsification of data throughout his career. Survey methods, meanwhile, suggest broad doubts within the science community^3^, with researchers estimating in one study that 17.1% of other researchers have falsified work^4^. While the true rate of false science is difficult if not impossible to detect^5^, the problem can only be more prevalent than the discovered cases.

Prior literature on retractions primarily examines biomedical journals, using PubMed data, and finds that retractions result in a 35–65% decrease in the retracted paper's citations compared to control papers^2^,^6^. Notably, citations to retracted papers do continue, and analyses suggest that half or more of the future citations continue to accept the original claims^2^,^6^,^7^,^8^,^9^,^10^,^11^. Thus, false results can live on, even after formal retraction, magnifying the consequences of publishing false results in the first place.

In this paper, we draw on all retraction notices in the Web of Science (WOS) database. We focus on the post-2000 period when WOS indexing of retractions appears relatively complete (see supporting information for detailed discussion of the database) and use the WOS to expand our analysis across the known universe of fields. Our analysis can thus provide a more comprehensive cross-field view of retractions than the existing literature. Most importantly, we examine a new dimension: We analyze the effect of retraction on scientists' *prior* work, thus quantifying a potentially critical consequence, and disincentive, for being associated with false scientific results. Our analysis further shows how chain reactions to retraction hinge on whether authors self-report errors.

## Results

**Figure 1:**
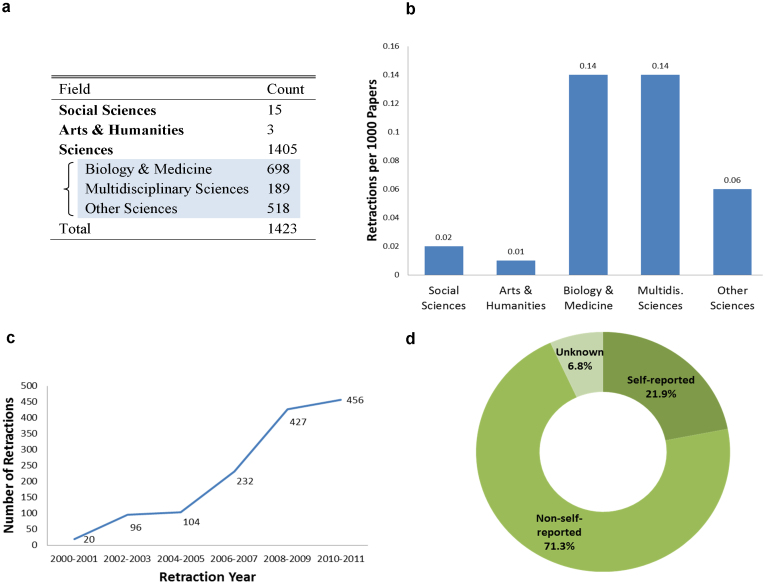
Retraction characteristics. Of the 1,423 retractions indexed by the Web of Science, the percentage of total retractions is greatest in the sciences, with nearly half (49.1%) of all retractions occurring in biology & medicine journals (a). Normalizing by field publication rates, both biology & medicine and multidisciplinary sciences show the greatest retraction tendency (0.14 papers per 1000 publications) (b). The number of retractions issued in a given period has been rising rapidly since the year 2000 (c). A minority (21.9%) of retractions is due to authors' self-reporting errors to the publishing journal (d).

Figure 1 presents basic characteristics of the retraction data. Retraction is most common in the hard sciences (Figs. 1a, 1b), especially in biomedical journals (.014% of biomedical papers) and multidisciplinary journals (.014% of papers), while occurring at approximately half this rate (.006% of papers) in other science fields. Meanwhile, social sciences (.002% of papers) and arts & humanities (.001% of papers) show substantially lower rates of retraction, which may reflect lower incidence of false science or lower rates of detection, where replication norms may differ^12^. Retraction rates are increasing with time (see also^13^ for PubMed analysis), measured by the year in which the retraction occurs (Fig. 1c). Based on the original publication year, retraction is more evenly distributed but still rising rapidly, with publications in 2008–2009 retracted at 2.3 times their rate in 2000–2001 (Table S1-1). Retraction is also substantially more frequent in the highest-impact journals. Papers published in Nature, PNAS, and Science are retracted at an average rate of 0.91% over the 2000–2009 period, which is 9.6 times the background retraction rate (Table S1-1), and retracted papers have higher average citations than non-retracted papers prior to retraction (Table S1-2). Among retracted articles, 312 retraction cases (21.9%) are “self-reported”, where the authors themselves report the error to the journal (Fig. 1d). However, the majority of cases are not self-reported, as further discussed in the supporting information (see also^13^). In sum, the problem of false science appears across many fields and at an increasing rate. Retractions are most likely among high-impact work and pre-eminent journals, and self-reported retractions are relatively rare.

Our empirical methodology compares the citation path of “treated” papers (those written by an author involved in a retraction) with the counterfactual citation path of “control” papers. Control papers are those that have similar citations paths to a treated paper prior to the retraction event. The effect of retraction is thus determined by examining the divergence, after retraction, between a treated paper and its ex-ante controls. As described below (see Methods), we use the entire WOS to find the most closely matched control papers within each field, allowing for substantially closer matches to the treated papers than can be determined using more limited databases.

**Figure 2:**
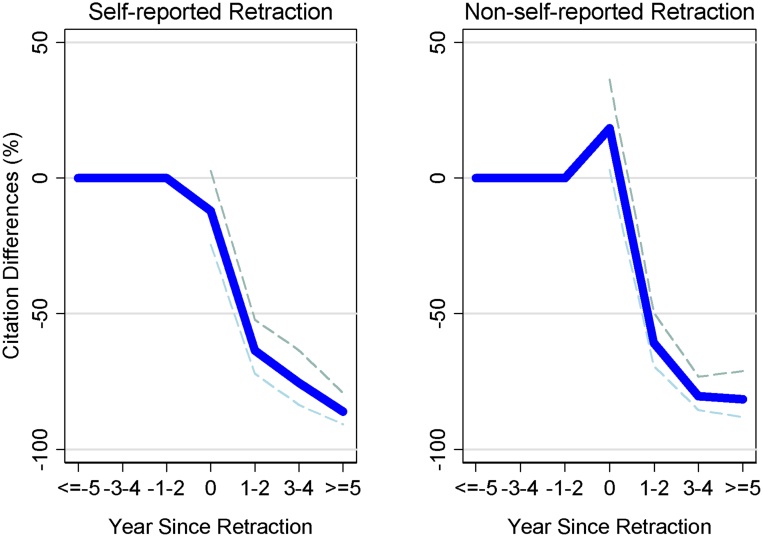
Effect of retraction on retracted papers themselves. Citations losses, compared to control papers, are shown for (a) self-reported retractions and (b) non-self-reported retractions. Blue lines indicate mean citation losses and dashed lines present 95% confidence intervals. Compared to the control papers, citation losses are 86.2% (p < .0001) for self-reported retractions and 81.5% (p < .0001) for non-self-reported retractions, annually, five or more years after retraction.

Figure 2 presents the effect of retraction on the retracted papers themselves. The effect appears similar for both self-reported retractions (Fig. 2a) and non-self-reported retractions (Fig. 2b), with the annual flow of citations five or more years after the retraction having dropped 86.2% (p < .0001) compared to the control papers for self-reported retractions and 81.5% (p < .0001) for the non-self-reported papers. The decline in citations to retracted work also appears broadly across scientific fields (Fig. S2).

**Figure 3:**
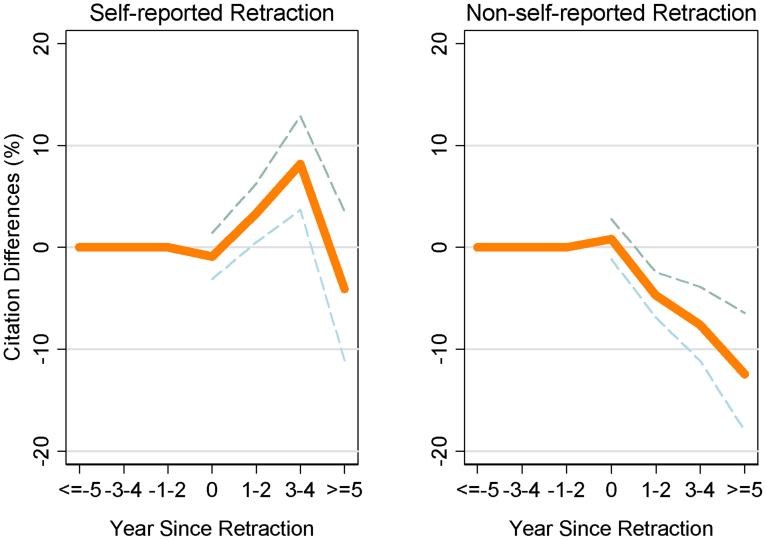
Effect of retraction on authors' prior body of work. Citations losses for prior work, compared to control papers, are presented after (a) self-reported retractions and (b) non-self-reported retractions. Orange lines indicate mean citation losses and dashed lines present 95% confidence intervals. After non-self-reported retractions, the authors' prior work loses 12.5% (p < .0001) of citations per year per prior publication five or more years after the retraction event, compared to control papers. By contrast, citation losses for the authors' prior body of work do not appear after self-reported retractions.

Figure 3 presents our main analysis, examining retraction effects on scientists' *prior* work. To isolate the effect of single retractions, we exclude cases where authors have multiple retractions, leaving 667 retracted papers and 1,737 authors with prior work. We build the sample of prior work using the WOS database. Specifically, we trace citations from each retracted article to prior articles by the same author (a 1^st^ degree self-citation), citations from these prior articles to other prior articles by the same author (a 2^nd^ degree self-citation), and so on up to the 11^th^ degree, at which point additional prior work is no longer revealed. We identify additional prior publications by tracing forward this citation network – locating papers by the same author that cite these past publications. The average number of prior articles per author generated is 25.9, creating a sample of 45,039 prior papers. Note that none of the prior work was itself retracted.

Figure 3 shows that retractions lead to substantial citation declines to the authors' prior work, but only if the retraction was *not self-reported*. When retractions are not self-reported, the annual flow of citations to a prior publication falls 4.7% (p < .0001) in the first two years after the retraction and 12.5% (p < .0001) five or more years after the retraction, compared to the control papers (Fig. 3b). Overall, the average loss after retraction is 6.9% (p < .0001), as shown in Table S2. By contrast, self-reported retractions do not show any statistically significant relationship to losses in citations to prior work, with point estimates suggesting essentially zero effect five years later and, if anything, increased citations at first (Fig. 3a). Thus, while retractions of both types have similar effect on the retracted paper itself, only retractions that are non-self-reported present large and sustained citations losses on the authors' prior body of work. Large citation losses to prior publications appear after non-self-reported retractions across the various sub-fields when analyzed separately, including biomedicine, multidisciplinary sciences, and other sciences (Fig. S3). Considering the smaller number of cases where an author experiences multiple retractions, as opposed to a single retraction, one sees more severe citation loss effects on prior work (Table S6).

The magnitude of the effect on prior work (Fig. 3) appears smaller than the effect on the retracted papers themselves (Fig. 2). However, recalling that authors on average have 25.9 prior papers, the prior publication penalty is in fact substantially magnified. In terms of linear citation counts (see Table S2), the average retracted paper loses 2.88 citations per year after retraction, compared to controls. A prior publication (for non-self-reported retractions) loses 0.091 citations per year on average, or 2.36 citations collectively (0.091 citations per paper × 25.9 papers per author), which is similar to the loss for retracted paper themselves. Moreover, five or more years after a non-self-reported retraction, the collective citation loss on an author's prior work rises to 5.39 citations per year (.208 × 25.9 papers, see Table S4-2).

**Figure 4:**
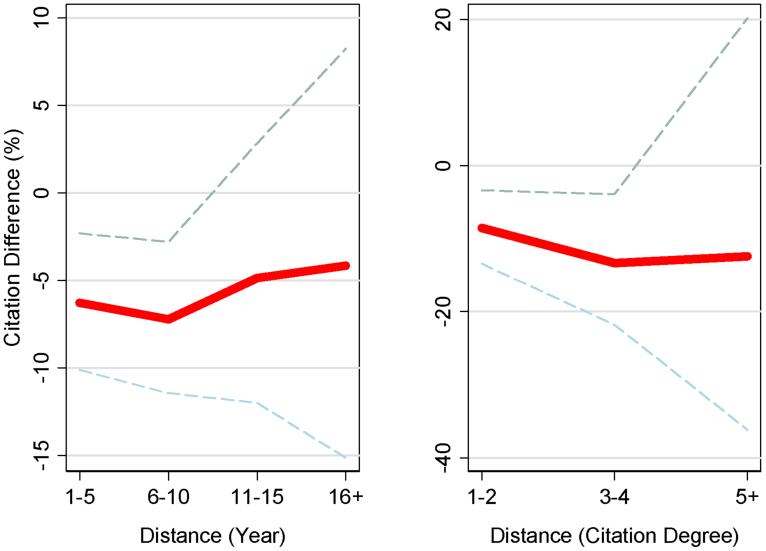
Effect on author's prior body of work by distance measures from retracted paper. Considering the effect of non-self-reported retractions, citation losses are sustained on the authors' prior work published up to 10 years before the retraction event (a), with negative but statistically insignificant losses for still earlier work. Citation losses on the authors' prior work are also sustained up to 4 degrees of separation away from the retracted paper in the author's citation network (b), with negative but statistically insignificant effects on work at higher degree of separation. Red lines indicate mean citation losses, and dashed lines present 95% confidence intervals. After many years, publications tend to have few annual citations, limiting the capacity for change vis-à-vis matched control papers and resulting in noisier estimates at high distance.

Focusing on non-self-reported retractions, Figure 4 examines the spillover effect on prior work, analyzing the distance between the retracted paper and the prior publications. Retraction spillovers remain negative and statistically significant for prior work published up to a decade earlier (Fig. 4a). For example, prior work published 6–10 years earlier sees citations fall 7.2% (p < .01) on average. The citation loss is similar in magnitude but not statistically significant for still older work. Examining citation losses by degrees of separation from the retracted paper (Fig. 4b), negative citation spillovers are found for papers up to four degrees of separation in the citation network (looking backwards in time). Prior publications three or four degrees of separation from the retracted paper experience citation declines of 14.3% (p < .01). The citation loss is similar in magnitude but not statistically significant for prior publications at five or more degrees of separation. Note that ongoing citations to older work are already low, which makes further declines difficult to estimate.

## Discussion

In sum, retractions can create substantial citations penalties well beyond the retracted paper itself. Citation penalties spread across publication histories, measured both by the temporal distance and the degrees of separation from the retracted paper. These broad citation penalties for an author's body of work come in those cases, the large majority, where authors do not self-report the problem leading to the retraction. By contrast, self-reporting mistakes is associated with no citation penalty and possibly positive citation benefits among prior work. The lack of citation losses for self-reported retractions may reflect more innocuous or explainable errors, while any tendency toward positive citation reactions in these cases may reflect a reward for correcting one's own mistakes.

These empirical findings are more broadly consistent with an informal policing mechanism among the scientific community, which reduces citations to the prior work of authors who are found to engage in a single instance of false science and fail to self-report. Fear of these broader penalties may discourage the publication of false results in the first place. Meanwhile, the opportunity to avoid them through self-reporting may encourage acknowledgment of mistakes, both helping to support, albeit imperfectly, core scientific norms regarding truth that stand at the center of scientific progress. Examining retraction effects on broader research activity in a field, the influence of retraction publicity and accusations of fraud, and differential effects across collaborators are all fascinating additional dimensions in this area of research^15^.

## Methods

In this paper, we draw on all retraction notices in the Web of Science (WOS) database. We focus on the post-2000 period when WOS indexing of retractions appears relatively complete and use the WOS to expand our analysis across the known universe of fields. Our analysis can thus provide a more comprehensive cross-field view of retractions than the existing literature.

To analyze retraction effects, we use a “treatment” and “control” methodology^2^,^10^. Treated papers are either the retracted papers themselves or, in our main analysis, prior publications by the same authors. Control papers are those with similar citation patterns to treated papers prior to the date of retraction. The control group generates the counterfactual comparison of what would typically happen to papers with similar initial citation patterns, had the retraction not occurred.

Control papers are those that minimize the distance from the treated papers' citation pattern prior to the retraction year. Specifically, define the set of papers in field *f* with publication year *p* as *N_fp_*. For a treated paper 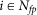, we search the WOS to find control papers 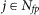 that minimize 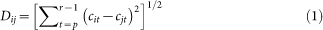
where *c_it_* denotes the citations paper *i* receives in year *t* and *r* is the year of retraction. Computationally, we define fields based on the 252 field categories in the WOS and locate, for each treated paper, ten control papers (with the same field and publication year as the treated paper) with the lowest *D_ij_*. In our main analysis, we take the two nearest neighbors, one from above and one from below the treated paper in terms of average citations prior to the retraction event. Because we access over 26 million articles in the WOS, this control strategy succeeds for the majority of treated papers (66.4%) in finding control paper pairs that on average have exactly the same citation pattern prior to the retraction event. We use this set for our primary analysis. The supporting information shows that the main results of the paper are robust to numerous alternative definitions of the control sample.

The regression calculates the decline in citations to treated papers, after the retraction event, compared to the counterfactual citation path of the control papers. Following standard methodology, we estimate regressions of the form 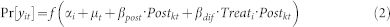
where the dependent variable, *y_it_*, is the number of citations received by paper *i* in year *t*. Fixed effects for each paper (*α_i_*) and each year since publication (*μ_t_*) capture the mean citation pattern of articles. *Treat_i_* is a dummy variable that equals 1 if article *i* is a treatment paper, and *Post_kt_* is a dummy variable that equals 1 if year *t* is after the retraction event for a given treatment and control group *k*. The coefficient of interest (*β_dif_*) captures any difference in citations for the treated paper, after the retraction event, compared to its control papers. In practice, we can use variations of^2^ to understand retraction effects for different periods after retractions, for different types of retractions, and for prior work that is more or less distant from the retracted paper. We estimate^2^ using the standard Poisson model for count data, given its robustness properties^14^. The supporting information shows that the results are robust to using many other regression models.

## Author Contributions

B.J. wrote the main manuscript text, S.L. prepared all figures and tables, G.J. wrote the supporting materials and B.U. prepared the data for analysis. All authors helped define the research questions and methodologies. All authors reviewed and edited the manuscript and Supplementary Information.

## Supplementary Material

Supplementary Information Supplementary Information
