# Supplementary material for: The Retraction Penalty: Evidence from the Web of Science
Source: Sci Rep. 2013 Nov 6;3:3146. doi: 10.1038/srep03146 (PMC3818648; doi:10.1038/srep03146)
Supplement: Supplementary Information [file srep03146-s1.pdf]

Supporting Information for

The Retraction Penalty: Evidence from the Web of Science

October 2013

Susan Feng Lu
Simon School of Business
University of Rochester
susan.lu@simon.rochester.edu

Ginger Zhe Jin
University of Maryland & NBER
ginger@umd.edu

Brian Uzzi
Kellogg School of Management
Northwestern University & NICO
uzzi@kellogg.northwestern.edu

Benjamin Jones*
Kellogg School of Management
Northwestern University & NBER
bjones@kellogg.northwestern.edu

This document describes the data sources, sample definition, econometric models, and robustness checks as cited in the paper.

1. Data sources

Our author, publication, citation, and citation network data come from the Web of Science (WOS) database collected by Thomson Reuters. Spanning 1945 to 2011, the WOS includes over 26 million journal articles published in over 10,000 of journals worldwide in all three major fields of scientific research: (1) science and engineering, (2) social sciences, and the (3) arts and humanities, which in aggregate include 252 subfields (physics, biology, sociology, architecture, English, etc.). The WOS provides information on citations of articles and the name, address and affiliation of authors. While the WOS is the largest known repository of scientific knowledge, it does not include every article in every subfield. For example, among the 4962 medical-specific journals covered in Medline, 57% are covered in WOS (http://science.thomsonreuters.com/news/2005-04/8272968/). The WOS's broad coverage of scientific publications implies that our analysis covers a nearly universal range of disciplines but cannot be fully exhaustive of papers in each discipline. The WOS also records retraction notices across the above fields.

Through an agreement with Thomson Reuters, our research group accesses the raw WOS directly, which is built into a database through 2009. With this database, we can perform analyses that are not possible using the publically available online system. In particular, we are able to build networks of prior publications by the authors of retracted articles, and we are also able to locate among the millions of indexed publications appropriate control papers with similar citation patterns to the authors' work (as discussed below).

WOS indexes retraction by inserting "retracted article" in the title of the original publication and "retraction of xxx" in the title of the retraction notice. Searching for "retracted article", "retraction article," and "retraction of" in the title for all articles published in the online WOS database as of August 31, 2011, we located 1,465 retracted articles and 1,614 retraction notices. Comparing the two lists, we found that some retraction notices retracted non-article type documents or articles that did not have full information in WOS. These cases were excluded from the count of retracted articles. Some retraction notices refer to articles included in WOS, but the original articles are not flagged by "retracted article" in the title. Adding these cases to the list of retracted articles and excluding retracted papers that were published after 2009, our sample of retractions included 1,423 original articles that had been retracted and have full records in the cleaned WOS database.

WOS began indexing retractions in 2003 and retroactively indexed retractions from earlier publication years. Given this timing, the WOS database does not appear comprehensive in early years (especially years before 2000). Hence, time trends in our sample appear accurately measured only in this past decade. Comparing retraction rates per publication in our analysis to

(13), which used PubMed data, we see similar retraction rates (and rises therein) in the last decade, while the PubMed database shows higher retraction rates prior to the 2000 than the WOS database. In practice, in either sample, retraction frequencies and rates are far higher in recent years.

For the 1,423 retracted articles, we classified the reasons for retraction by consulting the official retraction notices and other resources, such as Retraction Watch (www.retractionwatch.wordpress.com). There are many bases for retraction, such as plagiarism, data fabrication, failure to replicate, and author error. These classifications are not mutually exclusive and in many cases the retraction reason is not agreed upon (e.g. authors of the paper disagree) or the initially stated retraction reason may not be accurate upon further investigation (e.g. by the grant agency or authors' institutions), as shown by (13). We focused on a simple distinction that is usually unambiguously reported: whether or not the paper was retracted because the author(s) self-reported the error to the publishing journal. Note that while this approach enables objective classification, it cannot determine authors' underlying motives. The decision to self-report may include the desire to correct honest mistakes but it may also include cases where the author(s) have private information about misconduct, which they fear will otherwise come to light. To the extent that self-reporting signals honesty, or at least obfuscates misconduct, it might lead to differentially positive effects on how the science community interprets the same author's other work after the retraction. Note also that any systematic differences between self- and non-self-reported retractions that are constant across a retracted paper's history are adjusted for by our paper level and group level fixed effects in the regression analysis (see below). As reported in the paper, 312 of the 1423 retracted articles are coded "self-reported" in our data, and 1,014 of the 1423 are "non-self-reported." For a minority of cases (97 of 1423) we are unable to determine the retraction reason, despite extensive bibliographic research. Notably, the rate of self-reported retractions in our sample (21.9%) is very similar to the 21.3% rate of retractions due to error reported by (13).

When we study the effect on retracted papers, we use 1,085 of the 1,423 retracted papers only, because not all papers have the necessary information for that first analysis. In particular, 298 papers were retracted in the same year they were published, which makes matching with control papers implausible (see discussion of matching process further below). Meanwhile, 29 papers were published too recently to witness citation paper ex-post of retraction, and 11 papers do not have clear retraction years.

To study the spillover effect of retraction on prior work, we start by reintroducing the 298 papers that were retracted during the publication year (giving 1,383 retraction events to study). This larger sample is usable because the prior work was published some years before, which allows observation of pre-retraction histories for the prior work and allows matching to proceed. At the same time, when looking at prior work, we focus on authors with a single retraction only. This focus ensures that the spillover from a single retraction can be identified (rather than

contaminating the results with cases where the prior work is itself retracted). In focusing on authors with single retractions, the sample is limited to 667 retracted papers, which lead to 45,039 prior publications by authors of the retracted work. We discuss analysis of authors with multiple retractions further below.

Finally, we manually created a crosswalk between the field code in WOS and the discipline codes used in NSF and CASPAR[1] and classified fields into five major groups: (a) biology & medicine, which includes biological, medical and other life sciences, (e) multidisciplinary sciences, which includes multidisciplinary journals such as Nature, PNAS, and Science, (c) other sciences, which includes mathematics, physics, chemistry, engineering, earth and space sciences, agricultural sciences, and other science and technology areas, (d) social sciences, which includes economics, business, sociology, history, and other social studies, and (e) arts and humanities, which includes literature, poetry, dance, theater, film, and television production among other arts and humanities fields.

2. Sample definition

Our study design compares the citation patterns of "treated" and "control" articles. There are two types of treated articles. The first is the set of 1,423 retracted articles discussed above. The second is the set of prior publications by the same authors. Because multiple researchers may share the same name and the same author may change address and affiliation over time, we use citation links in the WOS database to identify prior work. In particular, we start with the articles cited in each retracted paper. Some of these cited articles share the same author name(s) as in the retracted article. We assume such a same-name author is the same author as in the retracted article and label these same-author articles as 1st degree self-citations. We then trace citations from these prior articles to other prior articles by the same author (a 2nd degree self-citation), and so on up to the 12th degree, at which point additional prior work is no longer revealed. All the above actions trace backward through the WOS. We also trace forward the citation network and further locate papers by the same author that cite these past publications. This process is repeated for every author of every retracted article until no more prior work can be found. By this definition, we capture prior work of the same authors that are directly or indirectly related to the retracted article. If an author published an article that is completely unrelated, or the publication is not covered by our WOS database, the article will not be counted as prior work. The average number of prior articles per author generated is 25.9, creating a sample of 45,039 prior papers.

Table S1-2 shows that, prior to their retraction, retracted papers have higher average citations than other papers. This tendency suggests that retracted papers are initially higher-profile papers. It also suggests that one cannot draw control papers at random within a field.

[1] See https://webcaspar.nsf.gov/nsf/srs/webcasp/data/gradstud.htm

Instead, one needs to carry out a matching process to locate control papers that share the same citation patterns with the treatment papers in the pre-retraction periods.

For a treated paper *i* published in field *f* and year p, we search for its control papers within the same field and the same publication year, where field is defined by the 252 field categories in the WOS. For each non-treated paper j in this pool, we define the arithmetic distance between *i* and j as

$$AD_{ij} = \sum_{t=p}^{r-1} (c_{it} - c_{jt})$$

and the Euclidean distance between i and j as:

$$ED_{ij} = \left[\sum_{t=p}^{r-1} \left(c_{it} - c_{jt}\right)^2\right]^{1/2}$$

where c_{it} denotes the citations paper *i* receives in year *t* and *r* is the year of retraction. Both distances attempt to measure the citation discrepancy between paper *i* and paper j, but arithmetic distance AD_{ij} indicates where j were cited less or more than *i* up to the year before retraction while Euclidean distance ED_{ij} is direction-free.

The quality of control group matching is assessed in Figure S1, which examines matches for authors' prior publications. A crude choice of control papers focuses on the ten papers with the lowest Euclidean distance to a treated paper. The upper-left graph of Figure S1 shows that the average Euclidean distance of the ten controls has high density around zero, which suggests close matches to the treated papers. In the bins for greater-than-zero distances, the density drops gradually except for the bin of 50 or more (which is driven by some retracted papers that were exceptionally highly cited). Because Euclidean distance is direction-free, there is no guarantee that the arithmetic distance of these ten control papers are distributed evenly on the two sides of the treated paper. As shown in the bottom-left graph of Figure S1, the average arithmetic distance of the ten controls has substantially more density on the negative side, so that these controls on average underestimate the citation flow of the treated papers.

If we restrict choice of control to only the one paper with the lowest Euclidean distance, we are able to find perfect match (with zero AD) for 39.7% of the treated papers. As shown in the bottom-middle graph of Figure S1, when we cannot find a perfect match, the arithmetic distance of the one control is still negative on average, though it is more evenly distributed on both sides of zero than the ten-control sample.

To achieve a more careful match, where control papers have low Euclidean distance and low average distance, we further consider the two nearest neighbors, one from above (with

positive AD) and one from below (with negative AD). As shown in the bottom-right panel of Figure S1, the density of the average arithmetic distance of these two controls is either exactly zero or concentrated in the neighborhood of zero. In particular, the two nearest neighbors now yield an average of zero distance for a substantially larger share (66.4%) of our treated papers. This sample, with zero average distance, is the main sample used in our analysis, as presented in the text. In this supporting information, we also consider a series of less conservative (but more inclusive) control strategies as robustness checks. The sample including all two-paper controls is labeled as two-control-full (all papers in the right-most panels in Figure S1). We also include an intermediate case including two-paper controls where the duration between retraction and publication is at least 2 years and where the average arithmetic distance is below 0.092 (the 95th percentile case, to limit outliers). This intermediate, refined sample is labeled as two-control-refined.

To summarize, we have constructed five alternative samples based on choice of control: two-control-zero (C2_zero), two-refined-control (C2_Refined), two-control-full (C2_full), one-control (C1), and ten-control (C10).

Table S1-1: Retraction Frequency by Retraction Year and Publication Year

	Retraction Year		Publication Year			
	Frequency		Frequency		Retraction per 1000 Papers	
	All Journals	Nature, PNAS, Science	All Journals	Nature, PNAS, Science	All Journals	Nature, PNAS, Science
2000-2001	20	4	132	29	0.055	1.739
2002-2003	96	25	150	7	0.060	0.419
2004-2005	104	14	242	27	0.089	1.535
2006-2007	232	20	400	9	0.134	0.500
2008-2009	427	15	319	7	0.127	0.428
2010-2011	456	14	29	1		
Mean					0.095	0.914

Note: There are 1.423 retractions in the WOS sample. Of these, 11 are not included in this table because the retraction year could not be determined and 77 occur prior to the year 2000.

Table S1-2: Retraction Frequency by Duration since Publication and Citation Impact

Duration until Retraction			Total Prior Citaitons (mean)		Prior Citations per Year (mean)	
Years	Number of Papers	Percentage of Papers	Retracted Papers	Non-Retracted Papers	Retracted Papers	Non-Retracted Papers
0	186	13.2	0	0	0	0
1	358	25.4	1	0	1	0
2	252	17.8	7	2	3	1
3	220	15.6	8	4	3	1
4	106	7.5	19	6	5	1
5	76	5.4	35	14	7	3
6	64	4.5	40	11	7	2
7	41	2.9	29	10	4	1
8	31	2.2	46	13	6	2
9	24	1.7	35	10	4	1
>=10	54	3.8	54	13	5	1
Total	1412	100.0	14	4	3	1

Note: There are 1.423 retractions in the WOS sample. Of these, 11 are not included in this table because the retraction year could not be determined.

Figure S1: Distributions of Different Samples

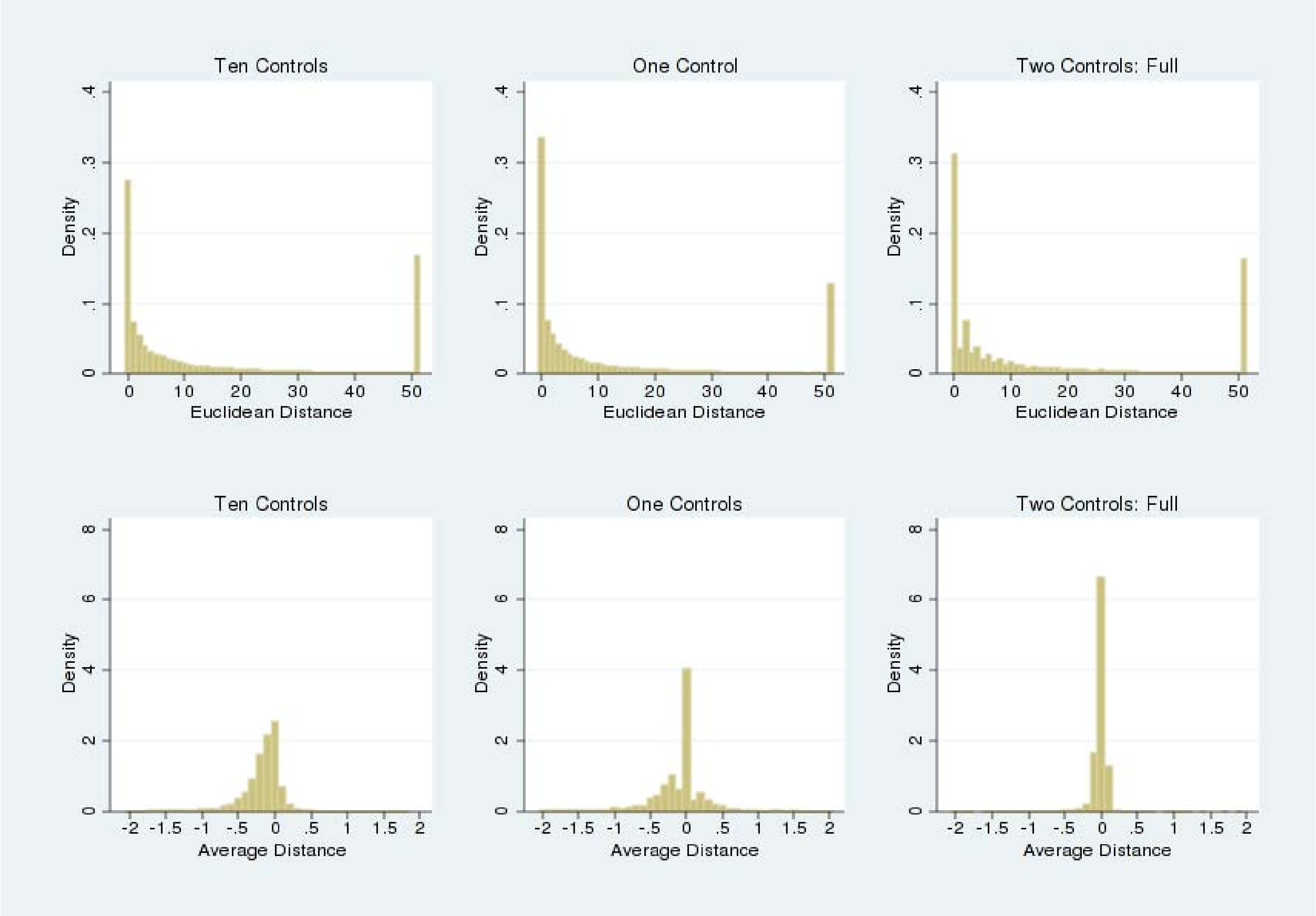

3. *Econometric models*

Our main dependent variable is the number of citations an article received in a particular year after publication. This variable is constructed by aggregating all the citation information in our WOS database. Because yearly citations are a form of count data (i.e. non-negative integers), we emphasize the Poisson model, given its robustness properties. However, we also consider the negative binomial model and classical ordinary least squares model (OLS).

Count models are estimated by maximum likelihood, based on a specification for the conditional mean of the count variable. Denote y_{it} as the number of citations that article i received in year t (since publication), $Post_{kt}$ as the dummy of whether t is after the retraction year for a given treatment and control group k that i belongs to, and $Treat_i$ as the dummy of whether i is a treated paper. The expected number of citations is defined as

$$E(y_{it}) = \exp\,(\alpha_i + \mu_t + \beta_{post} \cdot Post_{kt} + \beta_{dif} \cdot Treat_i \cdot Post_{kt}). \qquad \text{(S1)}$$

where fixed effects for each paper (α_i) capture the mean citation of articles and fixed effects for each year since publication (μ_t) capture the average citation pattern over years. The parameters are determined such that they maximize the overall likelihood of all observations.

The methodology of conditional maximum likelihood to estimate a Poisson model with fixed effects in panel data is developed in (16). More generally, (17) shows that the Poisson estimates are generally consistent as long as the conditional mean assumption (equation S1) is correct, making Poisson a conservative and robust estimator that imposes little structure on the underlying data generating process. While the consistency of Poisson estimates does not depend on any assumption on the variance of the count-data distribution, its standard error needs to be corrected for this generality. We correct the standard error of our Poisson estimates following (17). (Note that, in practice, this means that the Poisson estimate is consistent even when the variance and mean of the distribution are not equivalent, so that a Poisson estimator is not in fact imposing a Poisson distribution on the data for large samples.) The negative binomial model uses the same equation (1) for the mean of y_{it} but assumes y_{it} itself conforms to a negative binomial distribution, meaning that the estimator is not consistent if the count process is not negative-binomial. The negative binomial model also faces computational challenges in using large numbers of fixed effects. For these reasons, our main analysis uses Poisson, although we also present negative binomial models below.

Note that, because retractions happen at various points in the calendar year, we identify control papers (see Section 2 above) based on years strictly before the calendar year of retraction, and we identify the effects of retraction for years strictly after the calendar year of retraction. In the tables below, we thus decompose $Post_{kt}$ into $Post(t = 0)$ meaning the timing relative to

retraction is ambiguous, and $Post(t \geq 1)$ meaning t is strictly after the retraction year. Our analysis focuses on the coefficient β_{dif} in this post period $(t \geq 1)$, which cleanly estimates the effect of retraction by comparing the citations of treated papers compared to the counterfactual citation paths of the treated papers' controls.

OLS models provide simple alternatives to count models. OLS does not address the fact that yearly citations are strictly positive or integers, but it allows an extensive number of control variables and its estimation coefficient directly reflects the linear effect of retraction on citations (rather than the percentage effects revealed by count models). The simple OLS model can be written as:

$$y_{it} = \alpha_i + \mu_t + \beta_{post} \cdot Post_{kt} + \beta_{dif} \cdot Treat_i \cdot Post_{kt} + \varepsilon_{it} \qquad \text{(S2)}$$

where the error term is clustered at the treatment-control paper group level. A more sophisticated version of the OLS model takes each group of treated and control papers as the unit of observation and defines the citation difference between treated and control papers as the dependent variable. More specifically, let n_k denote the number of control papers in group k, we can write this "first-difference" model as:

$$\Delta y_{kt} = y_{it|Treat_i=1} - \frac{\sum_{j \in k} y_{jt|Treat_j=0}}{n_k} = \alpha_k + \mu_t + \beta_{dif} \cdot Post_{kt} + \varepsilon_{it.} \qquad \text{(S3)}$$

4. Main results and robustness checks

Focusing on single retractions and related prior work, Tables S2-S5 and Figures S2-S4 report our main results by samples, by regression models, by retracted and prior work, by duration, by citation degree, by broad disciplines, and by author order.

5. Results for multiple retractions

The analyses above focus on single retraction cases. For completeness, this section reports additional findings for multiple retractions. Authors with multiple retractions are a minority of cases (15% of authors with a retraction). In addition to the smaller sample size, these cases raise two technical difficulties for analysis. First, multiple retractions often happen over multiple years, so there is not a single event date to employ in the empirical strategy. Second, with multiple retractions, different retractions involving the same author may be self-reported and non-self-reported, making binary classification of the type less clear than in the single retraction cases. With these constraints in mind, one can perform analyses for multiple retraction cases by pooling the type of retraction and using the first retraction as the event date. The results are shown in column 1 of Table S6. We find that, for years $t \geq 1$, multiple retractions provoke similar mean citation losses to prior work as single retractions. However, multiple retractions also show a large, immediate effect in the year of retraction. This finding is further reinforced in

column (2), where we limit the multiple retraction cases to those authors where all their retractions happen in a single year. This sample specification allows for a single event date and a hence a more careful experimental design. The results shows increasingly negative point estimates for both the immediate and following citation losses. Overall, the greater immediate consequences and larger cumulative consequences for multiple retractions are consistent with the natural idea that multiple retractions will impose greater consequences for an individual than single retractions.

Table S2: Effects of Retraction on Retracted Papers and Prior work

	Retracted Papers						Prior Work					
	All Cases		Self-reported Cases		Non-self-reported Cases		All Cases		Self-reported Cases		Non-self-reported cases	
	Poisson	OLS	Poisson	OLS	Poisson	OLS	Poisson	OLS	Poisson	OLS	Poisson	OLS
	(1)	(2)	(3)	(4)	(5)	(6)	(7)	(8)	(9)	(10)	(11)	(12)
Treated*Post(t=0)	0.112**	0.527**	-0.111	-0.475	0.181**	0.791***	0.009	0.062***	-0.009	-0.023	0.008	0.084***
	(0.056)	(0.240)	(0.081)	(0.557)	(0.075)	(0.289)	(0.007)	(0.019)	(0.012)	(0.032)	(0.010)	(0.025)
Treated*Post(t≥1)	-1.090***	-2.881***	-1.240***	-3.591***	-1.189***	-3.037***	-0.038***	-0.029	0.031*	0.108***	-0.071***	-0.091***
	(0.104)	(0.412)	(0.154)	(0.768)	(0.139)	(0.586)	(0.011)	(0.018)	(0.017)	(0.031)	(0.015)	(0.023)
Year-Since-Publication Dummies	Y	Y	Y	Y	Y	Y	Y	Y	Y	Y	Y	Y
Paper Fixed Effects	Y	Y	Y	Y	Y	Y	Y	Y	Y	Y	Y	Y
Observations	16,118	18,507	4,447	4,686	10,080	11,967	999,262	1,044,486	371,188	384,852	558,703	587,517
R-squared		0.085		0.138		0.069		0.161		0.170		0.162

Standard errors are clustered by groups
*** p<0.01, ** p<0.05, * p<0.1

Using our most closely-matched sample (C2_zero, see Section 2 above), this table reports Poisson and simple OLS estimates for the impact of single retraction events on the retracted paper itself and on prior work. The estimation coefficients are reported, with standard errors in parentheses. The coefficient of Treated*Post(t>=1) represents the effect of retraction on the mean yearly citations of the treated paper (compared to controls) averaging across all years after retraction. For the Poisson model, the coefficient of Treated*Post(t>=1) can be translated into percentage terms as exp(coefficient)-1. For example, in column (1), the Poisson coefficient of Treated*Post(t>=1) implies that retraction reduces yearly citations of the retracted paper itself by exp(-1.090)-1= 66.4% (p<.0001). The OLS model provides results directly in lost citation counts. In column (2), the effect is seen as 2.88 fewer citations per year. Across all columns, Poisson estimates suggest that self-reported retractions lead to 71.1% (p<.0001) decline in the yearly citation of the retracted paper and 3.15% (p<.1) increase of citation to the prior work of same author(s). Non-self-reported retractions lead to a 69.6% (p<.0001) decline in the yearly citation of retracted papers and 6.85% (p<.0001) decline in citations to prior work.

Table S3-1: Effect of Retraction on Retracted Papers across Samples

	Self-reported Cases					Non-self-reported Cases				
Samples	C2_Zeros	C2_Full	C2_One_Retract	C1	C10	C2_Zeros	C2_Full	C2_One_Retract	C1	C10
	(1)	(2)	(3)	(4)	(5)	(6)	(7)	(8)	(9)	(10)
Treated*Post(t=0)	-0.128	-0.098	-0.07	-0.089	-0.119**	0.170**	0.101*	0.337***	0.139**	0.076
	(0.078)	(0.064)	(0.118)	(0.063)	(0.054)	(0.071)	(0.059)	(0.064)	(0.068)	(0.050)
Treated*Post(t=1/2)	-1.008***	-0.902***	-0.915***	-0.894***	-0.914***	-0.935***	-0.981***	-0.623***	-0.940***	-0.980***
	(0.137)	(0.110)	(0.159)	(0.103)	(0.095)	(0.126)	(0.104)	(0.140)	(0.101)	(0.098)
Treated*Post(t=3/4)	-1.406***	-1.309***	-1.324***	-1.284***	-1.363***	-1.627***	-1.728***	-1.243***	-1.771***	-1.792***
	(0.204)	(0.176)	(0.202)	(0.139)	(0.142)	(0.157)	(0.140)	(0.193)	(0.151)	(0.140)
Treated*Post(t>=5)	-1.977***	-1.887***	-1.929***	-1.902***	-1.957***	-1.687***	-1.802***	-1.264***	-1.934***	-1.798***
	(0.205)	(0.195)	(0.234)	(0.180)	(0.175)	(0.227)	(0.199)	(0.212)	(0.222)	(0.192)
Year-Since-Publication Du	Y	Y	Y	Y	Y	Y	Y	Y	Y	Y
Paper Fixed Effects	Y	Y	Y	Y	Y	Y	Y	Y	Y	Y
Observations	4,447	5,218	3,031	3,482	18,929	10,080	11,637	4,473	7,814	42,313

Standard errors in parentheses
*** p<0.01, ** p<0.05, * p<0.1

Using the Poisson model, this table compares the effect of retraction on retracted papers across different samples, including samples such as C1 and C10 that provide noisier matches with the treated papers. C2_One_Retract refers to the C2_full sample conditional on the 667 single-retractions only. The other columns draw on the 1,085 retracted papers that provide necessary information for the analysis (see discussion above). Treated*Post(t=1/2) refers to the effect of retraction in 1-2 years after retraction, similarly, Treated*Post(t=3/4) and Treated*Post(t>=5) refers to the effect of retraction in 3-4 years or 5-and-more years after retraction.

Table S3-2: Effect of Retraction on Prior Work across Samples

	Self-reported Cases					Non-self-reported Cases				
Samples	C2_Zeros	C2_Full	C2_Refined	C1	C10	C2_Zeros	C2_Full	C2_Refined	C1	C10
	(1)	(2)	(3)	(4)	(5)	(6)	(7)	(8)	(9)	(10)
Treated*Post(t=0)	-0.009	0.008	-0.018**	0.029**	0.019	0.008	0.033***	0.006	0.018*	0.047***
	(0.012)	(0.014)	(0.009)	(0.013)	(0.013)	(0.010)	(0.009)	(0.008)	(0.010)	(0.008)
Treated*Post(t=1/2)	0.033**	0.044**	0.001	0.073***	0.054***	-0.048***	-0.024**	-0.071***	-0.036***	-0.017
	(0.014)	(0.017)	(0.012)	(0.016)	(0.015)	(0.012)	(0.012)	(0.010)	(0.012)	(0.010)
Treated*Post(t=3/4)	0.079***	0.092***	0.057***	0.121***	0.105***	-0.079***	-0.029	-0.067***	-0.053**	-0.025
	(0.022)	(0.023)	(0.018)	(0.023)	(0.020)	(0.020)	(0.020)	(0.018)	(0.023)	(0.019)
Treated*Post(t>=5)	-0.041	0.049	0.013	0.072	0.042	-0.133***	-0.074***	-0.088***	-0.105***	-0.057**
	(0.039)	(0.050)	(0.032)	(0.049)	(0.041)	(0.034)	(0.028)	(0.029)	(0.035)	(0.026)
Year-Since-Publication Dummies	Y	Y	Y	Y	Y	Y	Y	Y	Y	Y
Paper Fixed Effects	Y	Y	Y	Y	Y	Y	Y	Y	Y	Y
Observations	371,188	710,171	649,830	473,576	2,602,439	558,703	1,037,092	951,650	691,598	3,798,735

Standard errors are clustered by groups
*** p<0.01, ** p<0.05, * p<0.1

Using the Poisson model, this table compares the effect on prior work across different samples, including samples such as C1 and C10 that provide noisier matches with the treated papers. All columns use 45,039 prior publications by authors of the retracted work, based on the 667 single-retraction cases. Treated*Post(t=1/2) refers to the effect of retraction in 1-2 years after retraction, similarly, Treated*Post(t=3/4) and Treated*Post(t>=5) refers to the effect of retraction in 3-4 years or 5-and-more years after retraction. Across samples, the effect of retraction after the retraction year is either zero or positive for self-reported cases; compared to 1-2 years after retraction, this effect increases slightly in 3-4 years after retraction but declines to close-to-zero in 5-and-more years after retraction. One potential explanation is that self-reporting is not only effective in separating the retracted paper from the authors' prior work, but also gives the authors and/or the prior work some positive exposure in a short period after the retraction. In comparison, the effect of non-self-reported retractions on prior work is significantly negative and persistent.

Table S3-3: Differential Effect of Retraction on Prior Work by Duration across Samples

	Non-self-reported Cases				
Samples	C2_Zeros	C2_Full	C2_Refined	C1	C10
	(1)	(2)	(3)	(4)	(5)
Treated*Post(t≥1)	-0.065***	-0.050**	-0.052***	-0.102***	-0.063***
	(0.021)	(0.022)	(0.020)	(0.025)	(0.019)
Treated*Post(t≥1)*Duration([6,10])	-0.010	0.018	-0.023	0.057*	0.029
	(0.032)	(0.029)	(0.028)	(0.033)	(0.026)
Treated*Post(t≥1)*Duration([11,15])	0.015	0.054	-0.037	0.113*	0.092*
	(0.045)	(0.056)	(0.036)	(0.061)	(0.050)
Treated*Post(t≥1)*Duration(>=16)	0.022	0.033	-0.024	0.153**	0.163***
	(0.066)	(0.046)	(0.040)	(0.062)	(0.053)
Year-Since-Publication Dummies	Y	Y	Y	Y	Y
Paper Fixed Effects	Y	Y	Y	Y	Y
Observations	558,703	1,037,092	966,634	691,598	3,798,735

Standard errors are clustered by groups
*** p<0.01, ** p<0.05, * p<0.1

Using the Poisson model, this table reports the effect on prior work by duration since publication, using different samples, including samples such as C1 and C10 that provide noisier matches with the treated papers. All columns use 45,039 prior publications by authors of the retracted work, based on the 667 single-retraction cases. Treated*Post(t>=1)*Duration(x,y) refers to the effect of non-self-reported retraction on prior work that has been published between x and y years at the time of the observation year t. All the C2 samples show no significant changes of the effect by duration. C1 and C10 show reduction of the effect for longer durations, probably because C1 and C10 have worse matches between treated and control papers than C2. The coefficients for duration 6-10, 11-15, and >=16 are relative to the default group of duration <=5.

Table S3-4: Differential Effect of Retraction on Prior Work by Citation Degree across Samples

	Non-self-reported Cases with Relevant Topics to Retracted Papers				
Samples	C2_Zeros	C2_Full	C2_Refined	C1	C10
	(1)	(2)	(3)	(4)	(5)
Treated*Post(t≥1)	-0.089***	-0.011	-0.062***	-0.048	-0.007
	(0.028)	(0.025)	(0.022)	(0.030)	(0.024)
Treated*Post(t≥1)*Degree(3/4)	-0.054	-0.096**	-0.095**	-0.041	-0.068*
	(0.060)	(0.041)	(0.039)	(0.043)	(0.041)
Treated*Post(t≥1)*Degree(5+)	-0.043	-0.067	-0.078	0.020	0.009
	(0.164)	(0.064)	(0.072)	(0.071)	(0.053)
Year-Since-Publication Dummies	Y	Y	Y	Y	Y
Paper Fixed Effects	Y	Y	Y	Y	Y
Observations	241,906	558,328	502,449	372,245	2,047,013

Standard errors are clustered by groups

*** p<0.01, ** p<0.05, * p<0.1

Using the Poisson model, this table reports the effect on prior work by citation degree, using different samples, including samples such as C1 and C10 that provide noisier matches with the treated papers. All columns are conditional on non-self-reported single retractions. Citation degree is measured by degree of separation from the retracted paper in the author's citation network, looking backward over time. The coefficients for degrees of 3-4 and degrees of 5+ are relative to the default group of degrees 1-2.

Table S4-1: Effect of Retraction on Retracted Papers across Alternative Specifications

	Self-reported Cases				Non-self-reported Cases			
Specifications	Poisson	OLS	First Difference	Negative Binomial	Poisson	OLS	First Difference	Negative Binomial
	(1)	(2)	(3)	(4)	(5)	(6)	(7)	(8)
Treated*Post(t=0)	-0.128	-0.474		-0.100	0.170**	0.783***		0.130*
	(0.078)	(0.555)		(0.091)	(0.071)	(0.290)		(0.068)
Treated*Post(t=1/2)	-1.008***	-4.133***		-1.003***	-0.935***	-3.171***		-0.947***
	(0.137)	(0.871)		(0.152)	(0.126)	(0.611)		(0.127)
Treated*Post(t=3/4)	-1.406***	-4.135***		-1.451***	-1.627***	-3.696***		-1.789***
	(0.204)	(1.061)		(0.225)	(0.157)	(0.837)		(0.166)
Treated*Post(t>=5)	-1.977***	-1.987***		-2.130***	-1.687***	-2.063***		-1.700***
	(0.205)	(0.723)		(0.250)	(0.227)	(0.615)		(0.207)
Post(t=0)			-0.474				0.783**	
			(0.597)				(0.320)	
Post(t=1/2)			-4.133***				-3.171***	
			(0.936)				(0.674)	
Post(t=3/4)			-4.135***				-3.696***	
			(1.141)				(0.924)	
Post(t>=5)			-1.987**				-2.063***	
			(0.777)				(0.679)	
Year-Since-Publication Dummies	Y	Y	Y	Y	Y	Y	Y	Y
Paper Fixed Effects	Y	Y	N	N	Y	Y	N	N
Group Fixed Effects	N	N	Y	N	N	N	Y	N
Treatment Dummy	N	N	N	Y	N	N	N	Y
Observations	4,447	4,686	1,562	4,686	10,080	11,967	3,989	11,967
R-squared		0.140	0.615			0.070	0.596	

Standard errors in parentheses clustered by group
*** p<0.01, ** p<0.05, * p<0.1

Using the C2_zero sample, this table shows that the effect of retraction on retracted papers is broadly robust to different econometric models. All columns use the 1,085 retracted papers that provide necessary information for the analysis (see discussion above). Note that computational constraints prevent inclusion of either paper or group fixed effects for the negative binomial model, weakening its identification of treatment effects.

Table S4-2: Effect of Retraction on Prior Work across Alternative Specifications

	Self-reported Cases				Non-self-reported Cases			
Specifications	Poisson	OLS	First Difference	Negative Binomial	Poisson	OLS	First Difference	Negative Binomial
	(1)	(2)	(3)	(4)	(5)	(6)	(7)	(8)
Treated*Post(t=0)	-0.009	-0.023		-0.001	0.008	0.085***		0.031***
	(0.012)	(0.032)		(0.012)	(0.010)	(0.025)		(0.010)
Treated*Post(t=1/2)	0.033**	0.126***		0.049***	-0.048***	-0.034		-0.013
	(0.014)	(0.037)		(0.015)	(0.012)	(0.027)		(0.012)
Treated*Post(t=3/4)	0.079***	0.200***		0.105***	-0.079***	-0.095**		-0.029
	(0.022)	(0.050)		(0.023)	(0.020)	(0.038)		(0.022)
Treated*Post(t>=5)	-0.041	-0.006		-0.096*	-0.133***	-0.208***		-0.072*
	(0.039)	(0.043)		(0.051)	(0.034)	(0.038)		(0.042)
Post(t=0)			-0.023				0.085***	
			(0.033)				(0.026)	
Post(t=1/2)			0.126***				-0.034	
			(0.039)				(0.029)	
Post(t=3/4)			0.200***				-0.095**	
			(0.052)				(0.040)	
Post(t>=5)			-0.006				-0.208***	
			(0.045)				(0.040)	
Year-Since-Publication Dummies	Y	Y	Y	Y	Y	Y	Y	Y
Paper Fixed Effects	Y	Y	N	N	Y	Y	N	N
Group Fixed Effects	N	N	Y	N	N	N	Y	N
Treatment Dummy	N	N	N	Y	N	N	N	Y
Observations	371,188	384,852	128,284	384,852	558,703	587,517	195,839	587,517
R-squared		0.171	0.405			0.163	0.370	

Standard errors in parentheses clustered by group
*** p<0.01, ** p<0.05, * p<0.1

Using the C2_zero sample, this table shows that the effect of retraction on prior work is broadly robust to different econometric models. All columns use 45,039 prior publications by authors of the retracted work, based on the 667 single-retraction cases. Note that computational constraints prevent inclusion of either paper or group fixed effects for the negative binomial model, weakening its identification of treatment effects.

Table S4-3: Effect of Retraction on Prior Work by Duration across Alternative Specifications

	Non-self-reported Cases			
	Poisson	OLS	First Difference	Negative Binomial
	(1)	(2)	(3)	(4)
Treated*Post(t≥1)	-0.065***	-0.123**		0.003
	(0.021)	(0.060)		(0.030)
Treated*Post(t≥1)*Duration([6,10])	-0.010	0.015		-0.102**
	(0.032)	(0.070)		(0.042)
Treated*Post(t≥1)*Duration([11,15])	0.015	0.093		-0.055
	(0.045)	(0.065)		(0.052)
Treated*Post(t≥1)*Duration(>=16)	0.022	0.110*		-0.037
	(0.066)	(0.062)		(0.074)
Post(t≥1)			-0.123**	
			(0.063)	
Post(t≥1)*Duration([6,10])			0.015	
			(0.073)	
Post(t≥1)*Duration([11,15])			0.093	
			(0.068)	
Post(t≥1)*Duration(>=16)			0.110*	
			(0.065)	
Year-Since-Publication Dummies	Y	Y	Y	Y
Paper Fixed Effects	Y	Y	N	N
Group Fixed Effects	N	N	Y	N
Treatment Dummy	N	N	N	Y
Observations	558,703	587,517	195,839	587,517
R-squared		0.165	0.370	

Standard errors in parentheses by group
*** p<0.01, ** p<0.05, * p<0.1

Using the C2_zero sample, this table shows that the differential effect of retraction on prior work by duration is robust to different econometric models. All columns are conditional on non-self-reported single retractions. Note that computational constraints prevent inclusion of either paper or group fixed effects for the negative binomial model, weakening its identification of treatment effects.

Table S4-4: Effect of Retraction on Prior Work by Citation Degree across Alternative Specifications

	Non-self-reported Cases with Relevant Topics to Retracted Papers			
	Poisson	OLS	First Difference	Negative Binomial
	(1)	(2)	(3)	(4)
Treated*Post(t≥1)	-0.089***	-0.250***		-0.068*
	(0.028)	(0.075)		(0.037)
Treated*Post(t≥1)*Degree(3/4)	-0.054	0.112		-0.058
	(0.060)	(0.092)		(0.068)
Treated*Post(t≥1)*Degree(5+)	-0.043	0.206**		-0.059
	(0.164)	(0.094)		(0.193)
Post(t≥1)			-0.260***	
			(0.076)	
Post(t≥1)*Degree(3/4)			0.125	
			(0.093)	
Post(t≥1)*Degree(5+)			0.218**	
			(0.095)	
Year-Since-Publication Dummies	Y	Y	Y	Y
Paper Fixed Effects	Y	Y	N	N
Group Fixed Effects	Y	Y	Y	N
Treated Dummy	N	N	N	Y
Observations	241,906	242,538	80,846	242,538
R-squared		0.218	0.266	

Standard errors in parentheses by group
*** p<0.01, ** p<0.05, * p<0.1

Using the C2_zero sample, this table shows that the differential effect of retraction on prior work by citation degree is robust to different econometric models. All columns are conditional on non-self-reported single retractions. Citation degree is measured by degree of separation from the retracted paper in the author's citation network, looking backward over time. The coefficients for degrees of 3-4 and degrees of 5+ are relative to the default group of degrees 1-2. Note that computational constraints prevent inclusion of either paper or group fixed effects for the negative binomial model, weakening its identification of treatment effects.

Table S5: Effect of Retraction on Prior Work, by Author Position in Retracted Paper

	Non-self-reported Cases		
	First Author	Middle Author	Last Author
	(1)	(2)	(3)
Treated*Post(t=0)	0.020	0.011	0.001
	(0.037)	(0.018)	(0.013)
Treated*Post(t≥1)	-0.118***	-0.091***	-0.054***
	(0.045)	(0.027)	(0.019)
Year-Since-Publication Dummies	Y	Y	Y
Paper Fixed Effects	Y	Y	Y
Observations	42,187	199,537	308,204

Standard errors in parentheses clustered by groups
*** p<0.01, ** p<0.05, * p<0.1

Using the C2_zero sample and the Poisson model, this table shows the effect of retraction on citations to the prior work of the authors, dividing the authors sample into three groups depending on whether they were the first, last, or a middle author on the retracted paper. All columns are conditional on non-self-reported single retractions.

Table S6: Effect of Retraction on Prior Work, Authors with Multiple Retractions

	All cases	Same Year
	(1)	(2)
Treated*Post(t=0)	-0.072***	-0.093***
	(0.015)	(0.022)
Treated*Post(t≥1)	-0.057**	-0.076**
	(0.025)	(0.035)
Year-Since-Publication Dummies	Y	Y
Paper Fixed Effects	Y	Y
Observations	337,522	207,173
Number of Paper id	28,297	17,127

Standard errors in parentheses clustered by group
*** p<0.01, ** p<0.05, * p<0.1

Using the C2_zero sample and the Poisson model, this table shows that the citation losses to prior work for authors who have 2 or more retractions. The event date is taken as the date of the first retraction. Column (1) considers all multiple retraction cases, while column (2) considers only those multiple retraction cases where all of the author's retractions occurred in the same year.

Figure S2: Effect of Retraction on Retracted Papers by Fields

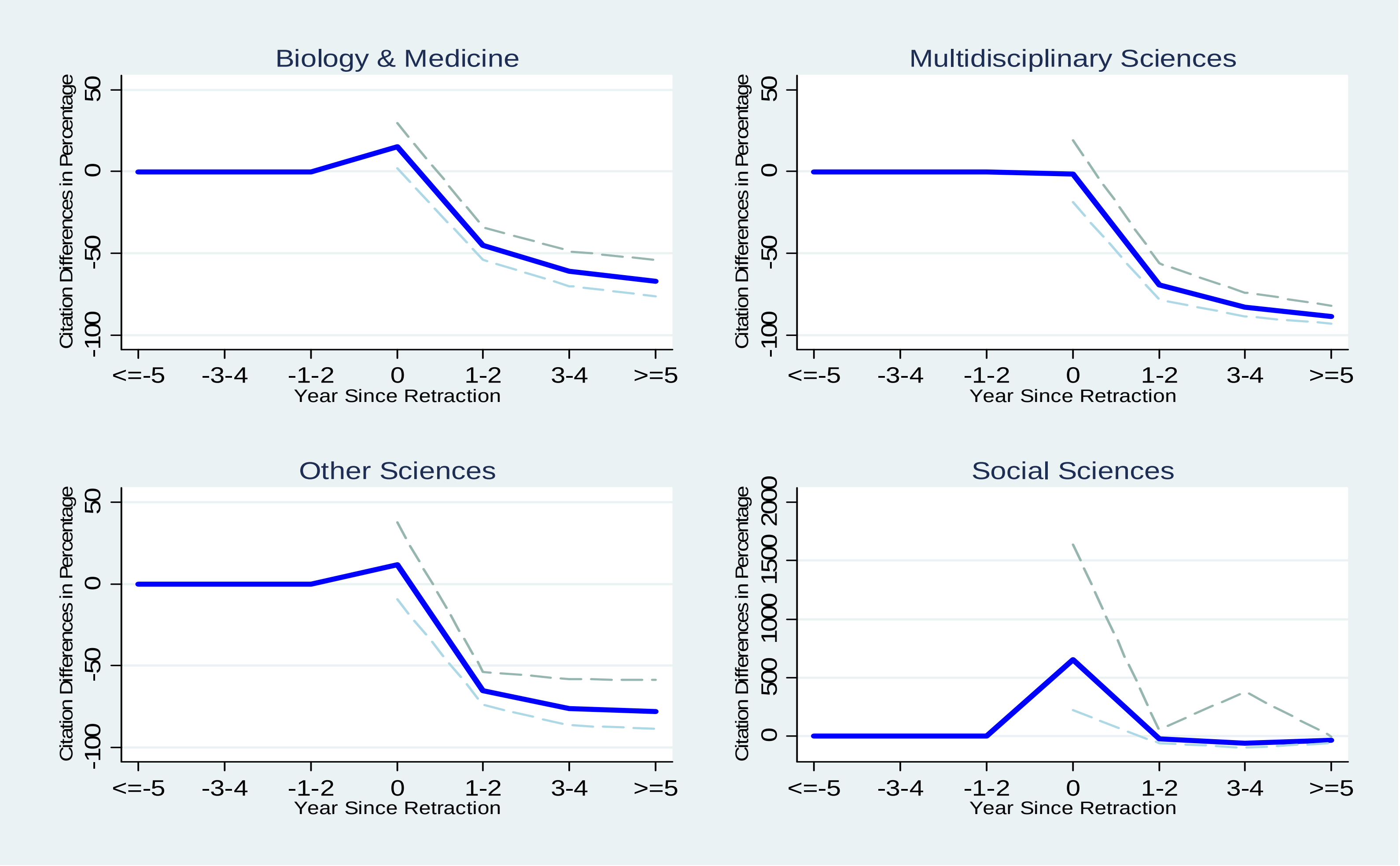

Using the C2_zero sample and the Poisson model, this figure plots the effect of retraction on retracted papers over time by broad disciplines. Dashed lines show the 95% confidence interval. Accurate inference for Social Sciences is difficult because there are only 15 such retractions in the sample.

Figure S3: Effect of Retraction on Prior Work by Fields, Non-Self-Reported Cases

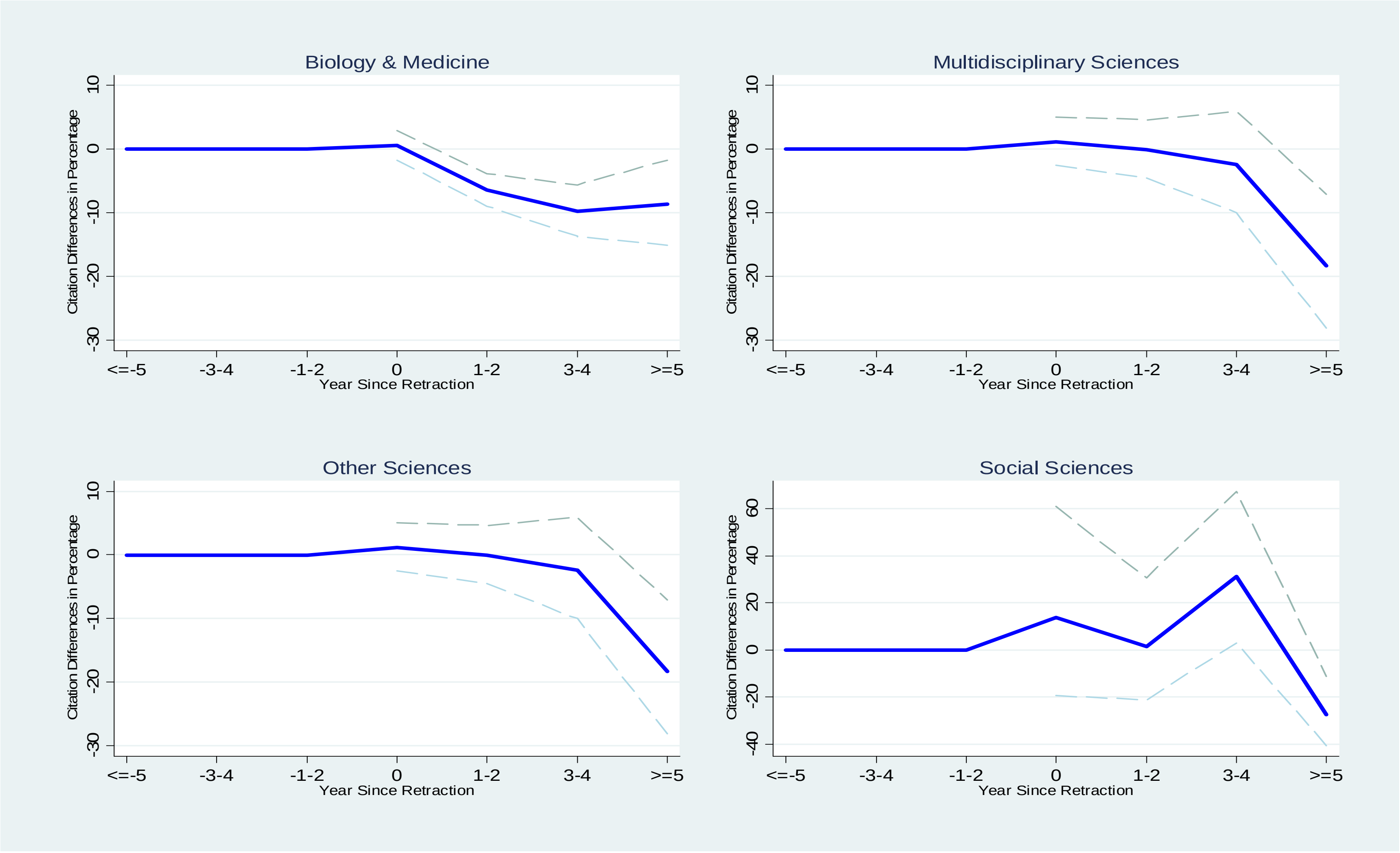

Using the C2_zero non-self-reported sample and the Poisson model, this figure plots the effect of retraction on prior work over time by broad disciplines. Dashed lines show the 95% confidence interval. Inference for Social Sciences is more challenging, due to fewer observations in this case.

Figure S4: Main Results, Further Disaggregating Effects

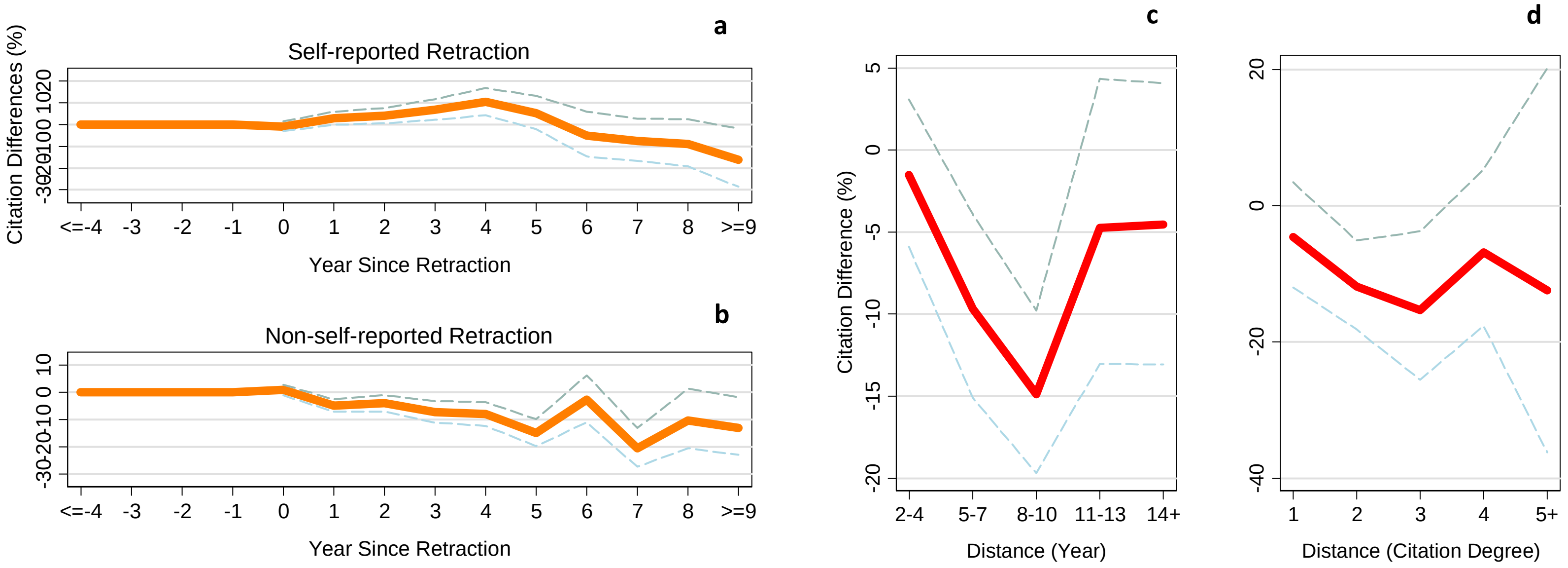

The results in Fig. 3 (main text) are further disaggregated by individual years since retraction (**a** and **b**). The results in Fig. 4 (main text) are further disaggregated by duration since publication of prior work (**c**) and network degree distance (**d**).
